# Impact of Pediatric Hematopoietic Stem-Cell Transplantation on Craniofacial Growth

**DOI:** 10.6061/clinics/2020/e1901

**Published:** 2020-10-21

**Authors:** Alexandre Viana Frascino, Marcelo Fava, Maria Dulce Silveira Collassanti, Vicente Odone-Filho

**Affiliations:** I Instituto da Crianca (ICr), Hospital das Clinicas (HCFMUSP), Faculdade de Medicina, Universidade de Sao Paulo, SP, BR; II Instituto de Ciencias Tecnologicas (ICT), Universidade Estadual de Sao Paulo (UNESP), Sao Jose dos Campos, SP, BR

**Keywords:** Hematopoietic Stem-Cell Transplantation, Pediatrics, Bone Marrow Transplantation, Skull/Growth and Development, Facial Bones/Growth and Development

## Abstract

**OBJECTIVES:**

To assess the craniofacial skeletal growth in pediatric hematopoietic stem-cell transplantation (HSCT) survivors in comparison with age-sex matched-paired controls.

**METHODS:**

A case-controlled retrospective comparison of the craniofacial growth in 25 HSCT children and 25 matched-paired controls was conducted. Craniofacial growth was quantitatively assessed by linear and angular measurements in panoramic radiographic images using ImageJ^¯^. Stature growth and body weight were obtained through physical examination. Cancer diagnosis, myeloablative conditioning, and HSCT were retrieved from medical records.

**RESULTS:**

Patients aged 12.2 years (±3.8; 16 male, 9 female). Radiographic images were obtained on an average of 2.43 (±2.0) years after HSCT. The main malignant diagnosis was acute lymphoblastic leukemia (56%), followed by acute myeloid leukemia (36%) and myelodysplastic syndromes (8%). Total body irradiation was associated with chemotherapy at 80%. Mean age at transplantation was 10 (±4.7) years. HSCT survivors showed reduced a vertical growth of the mandibular ramus (*p*=0.003). This persisted among individuals below 12 years of age (*p*=0.017). The HSCT group showed delayed dental eruption, though there was no statistically significant difference (*p*=0.3668). The HSCT group showed stature deficit, increased weight, and body mass index (Z-score stature: -0.28; Z-score weight: 0.38, respectively).

**CONCLUSIONS:**

Pediatric HSCT has decreased vertical craniofacial growth compared to their matched controls. There might be an association between reduced craniofacial vertical growth and reduced estature growth. Further studies to quantitatively investigate the impact of different myeloablative regimens in craniofacial skeletal growth and development.

## INTRODUCTION

Hematopoietic stem-cell transplantation (HSCT) has become one of the most important approaches for pediatric patients with onco-hematological malignancies (1-3). HSCT is preceded by intensive myeloablative conditioning, isolated or combined total body irradiation, and chemotherapy (4). It is estimated that 10,000 transplants are performed annually worldwide (5).

Although HSCT for pediatric patients has been associated with increased survival rates, several long-term complications have been reported (6,7). Endocrine, cardiopulmonary, gastrointestinal, hepatic, renal, neurological, and skeletal impairments clinically manifest as low stature, disproportional growth, osteoporosis, increased risk of bone fractures, diabetes mellitus, delayed sexual maturation, and cognitive deficit (8,9).

Impaired skeletal growth and development have been associated to reduce bone mineral density in the femur, vertebrae, and jaws (10-13). Craniofacial and dental development disturbances have been described to be more prevalent in children submitted to HSCT at a young age (<10 years) (14-16,). Impaired skeletal growth in long-term childhood HSCT survivors negatively affects their quality of life (6,12,13).

The present retrospective study aimed to quantitatively assess craniofacial growth in HSCT children in comparison with age-sex matched-paired controls.

## MATERIAL AND METHODS

Between 2015 and 2018, long-term pediatric HSCT survivors were selected from Instituto do Tratamento do Câncer Infantil, Instituto da Criança, Hospital das Clínicas da Faculdade de Medicina da Universidade de São Paulo.

The case group (HSCT) inclusion criteria were as follows: 1) HSCT for the treatment of hematological malignancies; 2) age at the time of HSCT ≤18 years; and 3) panoramic radiograph taken at least 6 months after HSCT. The exclusion criteria were as follows: 1) diagnosis of skeletal disorders; 2) previous orthodontic treatment; 3) the presence of orthodontic appliances, and 4) history of craniofacial trauma. The control group (CONTROL) individuals were selected matched by age-sex from Instituto de Ciências e Tecnologia, Faculdade de Odontologia, UNESP - São José dos Campos - São Paulo - Brazil.

Information regarding date of birth, sex, height, weight, medical history, and myeloablative conditioning regimen was retrieved from the medical records. Oral and maxillofacial health information was retrieved from the dental records.

Panoramic radiographs were obtained at Instituto de Radiologia, InRad-HC-FMUSP, using the Orthophos CD (Siemens, Bensheim. Germany) with imaging settings of 60-90 kVp, 9-12 mAs and 12 s of exposure. All radiographic images were downloaded with 256 gray levels, 3188×1709 pixels, and 300 dpi resolution in digital format (JPEG) compatible with Image J (1.50c4 for Mac OS Sierra 10.12.6).

**Figure 1:**
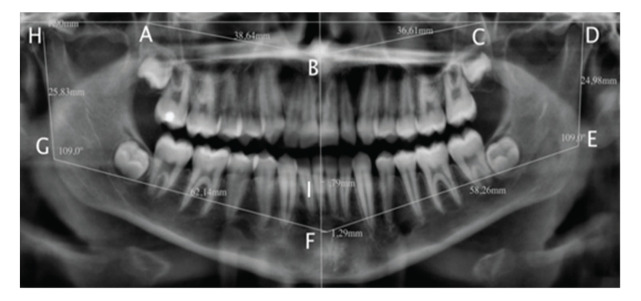
Maxillomandibular cephalometric assessment. **A.** posterior side of the right jaw; **B.** anterior nasal spine; **C.** posterior side of the left maxilla; **D.** head of the left jaw; **E**. left mandibular angle; **F.** mentonian fossa; **G.** right mandibular angle; **H.** right jaw head; **I.** apex of the inferior interincisal alveolar ridge.

Craniofacial growth was assessed radiographically using linear and angular distances between predetermined topographic anatomical points in the jaws (Figure 1) (17). Two blinded observers were previously trained for conducting cephalometry studies. The dental age was estimated by the Nolla tooth development stage (18).

The sample size was estimated before data collection based on a review of previous studies (10,13,14). A primary error probability of 5% and statistical power of 80% was assumed. Statistical comparisons were undertaken through the Student t-test using Excel for Mac (Microsoft version 15.37).

This study was approved by the Research Ethics Committee of the Faculdade de Medicina, Universidade de São Paulo (CEP/CONEP number 05139018.9.0000.0068). The parents or legal guardians of the participants provided written consent for their children to participate in the study.

## RESULTS

### Patients Characteristics and Oncologic Treatment

Fifty panoramic radiographic images were analyzed (25 HSCT in the group and 25 CONTROL in the group). Radiographic images were obtained on an average of 2.43 (±2.0) years after HSCT.

Age and sex of the control group were matched to the study group. Sixteen male and nine female patients composed each group. The mean age at transplantation was 10 (±4.7) years. The mean age during radiography was 12.2 (±3.8) years.

Fifty-six percent of the primary cancer diagnosis was acute lymphoblastic leukemia, 36% was acute myeloid leukemia, and 8% was myelodysplastic syndromes. Individualized primary malignant diagnosis has been described in Supplementary file 1.

Pre-HSCT myeloablative conditioning included different combinations of cyclophosphamide (76%), fludarabine (40%), busulfan (20%), melphalan (20%), and etoposide (8%). Eighty percent of the included patients (n=20) received myeloablative pre-HSCT total body irradiation divided into six sessions of 200 cGy, totaling 1200 cGy for each patient. All patients received methotrexate and corticosteroids.

In 21% of the cases, autogenous HSCT was performed and the remaining were allogeneic. Of these, 36.3% received stem cells from related donors, 36.3% from unrelated donors, and 27.2% from the umbilical cord. The mean age during HSCT was 10 (±4.7) years.

### Height and Weight Assessment

Younger (age<12 years) male and female HSCT patients were below the stature average and had above-average weight for their age (Z-score_ height_: -0.13; Z-score_ weight_: +1.8). In older patients (age>12 years), this was seen in the male-only comparison (Z-score _height_: -1.5; Z-score_ weight_: +0.68). Female patients showed decreased average stature and weight (Z-score _height_: -1.4; Z-score _weight_: -0.95). Table 1 shows the absolute height and weight mean values, body mass index (BMI), and SD (Z-score) for each age group.

### Cephalometric Assessment

HSCT group showed a smaller vertical growth in the mandibular ramus with significant statistical differences (*p*=0.003). The comparison of individuals younger than 12 years did not show significant difference (*p*=0.13). The comparison of individuals above 12 years of age showed a significant reduction in the mandibular ramus vertical growth (*p*=0.017). Table 2 presents vertical extensions and the mean values of mandibular ramus for both groups.

There were no statistically significant differences in the comparison of the other linear or angular cephalometric assessments. Table 3 presents the mean values for both groups.

### Dental age estimation

**Figure 2:**
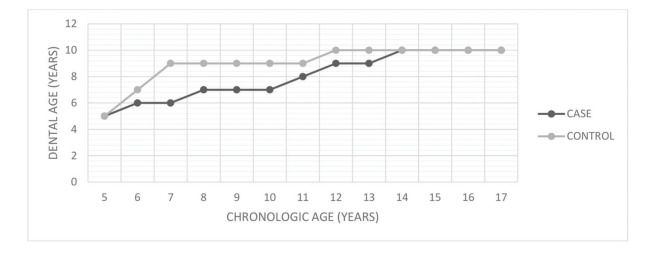
Dental chronological age. Dental chronological age estimation based on tooth root formation.

HSCT patients showed delayed dental formation and eruptive chronology, though there was no statistically significant difference between the groups (*p*=0.3686). Figure 2 presents the correlation between chronological and dental age according to the dental maturation stage (18).

## DISCUSSION

HSCT plays an important role in the management of hematologic high-risk pediatric malignancies with elevated long-term disease-free survival rates (1-3). However, associated late complications include craniofacial impairment and low stature (10,19).

In this study, we found reduced vertical growth in the mandibular ramus of HSCT patients in comparison to healthy controls (*p*=0.003). This difference persisted in patients older than 12 years (*p*=0.017), but was not seen in the younger group (<12 years, *p*=0.13), suggesting that HSCT plays an active role lowering stature and impairing the craniofacial skeleton. Previous reports indicate a positive correlation between somatic and craniofacial growth (20). In addition, the observed statistically significant differences that persisted in the >12 year-old group suggest that the puberal spurt does not lead to the catch-up growth of craniofacial bones (21). HSCT patients showed late dental eruption compared to their matched controls. Dental eruption was previously assumed to be a causative effect of reduced vertical mandibular growth in early age HSCT survivors (16). However, further studies are required to establish a positive correlation between delayed dental eruption and reduced craniofacial vertical growth.

Craniofacial changes are associated with increased risk of sleep apnea, respiratory disorders, and impaired neurodevelopment (22). The interpretation of our results allows us to infer that the vertical deficit of the face observed in our results may be a contributing factor to the development of respiratory diseases during adulthood, and further studies with this population are needed.

The early detection of craniofacial deformities is important to provide childhood HSCT survivors proper care and mitigate the quality of life impairment associated with oncologic treatments. Panoramic radiographs present as a trustworthy and cost-effective tool for craniofacial growth screening early malocclusion diagnosis (23,24).

The post-transplantation period of evaluation (mean 2.43 years, min.: 6 months; max.: 7 years) did not considerably affect the comparative analyses of craniofacial growth for the following reasons. First, patients aged below 12 years undergoing HSCT have a significantly higher risk for dental and jaw aberrations compared with healthy individuals. Second, the age at transplant has a higher impact on dental development and craniofacial growth compared with conditioning regimens.

The main limitation of this study was the impossibility of separately studying the different myeloablative regimens due to the high individualization of chemotherapeutic agents to minimize the side effects. However, this limitation does not invalidate the present results since no differences were observed between the prevalence of dental anomalies and different myeloablative chemo-radiotherapy protocols (14).

The comparative analysis of craniofacial growth and development showed statistically significant differences in the group of patients over 12 years of age. These results suggest that unwanted HSCT effects include late craniofacial skeletal changes. However, more long-term studies are needed to study these variables.

## CONCLUSIONS

Compared to the control group, pediatric HSCT patients had delayed skeletal changes due to impaired craniofacial growth and development.

Statistically significant differences were observed in the growth of the Madibular Ramus, which was maintained in individuals over 12 years of age.

## APPENDIX

## AUTHOR CONTRIBUTIONS

Frascino AV contributed in writting of the article. Collassanti MD contributed in HSCT and oncological data. Fava M contributed in Radiographic and dental suppervision. Odone-Filho V contributed in Main suppervision, text review.

## Figures and Tables

**Table 1:** Height and weight mean values, BMI, and SD (Z-score).

	Sex	Mean age (years)	Weight (kg); Z-score	Height (cm); Z-score	BMI; Z-score
Age <12 years	Male	8	34±1.8	127±0.13	21±2.5
	Female	7	23±0.18	116±0.88	17±0.91
Age >12 years	Male	14	48±0.68	152±1.5	20±0.68
	Female	14	39±0.95	150±1.4	17±0.95

BMI: body mass index, is a measure of body fat based on height and weight; SD: Standard deviation; Z-score: SD below or above the population mean raw score.

**Table 2:** Mandibular ramus vertical extension (mm).

	HSCT group				Control group				*p*
	*N*	*Mean*	*Median*	*SD*	*N*	*Mean*	*Median*	SP	
Younger group (age <12 years)	14	32.90	32.24	4.84	12	39.46	43.55	5.20	**0.130**
Older group (age >12 years)	11	43.46	43.96	7.17	13	48.57	49.70	8.68	**0.017** *
Total	25	40.92	41.73	8.05	25	46.75	48.03	8.77	**0.003** *

* *p*<0.05. SD: standard deviation. The bold numbers are the statistical significant difference between case-study groups.

**Table 3:** Cephalometric maxillomandibular assessment.

		HSCT group	Control group	*p*
**1**	**A-B**	48.10 m	48.29 mm	**0.68**
**2**	**B-C**	50.09 mm	50.03 mm	**0.67**
**3**	**H-G**	40.32 mm	45.98 mm	0.004*
**4**	**D-E**	40.53 mm	46.51 mm	0.002*
**5**	**G-F**	81.74 mm	81.92 mm	**0.93**
**6**	**E-F**	81.41 mm	81.36 mm	**0.94**
**7**	**F-I**	1.25 mm	1.43 mm	**0.57**
**8**	**HGF**	110.00°	110.12°	**0.55**
**9**	**DEF**	109.48°	110.56°	**0.65**

A-B: right maxilla; B-C: left maxilla; H-G: right mandibular ramus; D-E: left mandibular ramus; G-F: right mandibular body; E-F: left mandibular body; F-I: anterior mandibular alveolar ridge; HGF: right mandibular angle; DEF: left mandibular angle.

* *p*<0.05. The bold numbers are the statistical significant difference between case-study groups.

## APPENDIX

**Supplementary file 1:** Individual malignant diagnosis.

ID	SEX	AGE AT HSCT(Y)	MALIGNANT DIAGNOSIS
1	female	2.3611	AML
2	male	3.4473	AML
3	female	4.6785	ALL
4	male	6.5116	ALL
5	male	6.7852	AML
6	female	7.4145	MDS
7	male	7.6936	ALL
8	female	7.6936	ALL
9	male	8.4323	ALL
10	female	8.4432	AML
11	male	8.4706	ALL
12	female	9.554	MDS
13	female	9.554	ALL
14	male	10.202	ALL
15	male	10.492	ALL
16	male	11.083	ALL
17	male	11.644	ALL
18	male	12.252	AML
19	male	13.576	AML
20	female	13.953	AML
21	female	14.367	AML
22	male	14.547	ALL
23	male	14.843	AML
24	male	15.141	ALL
25	male	15.157	ALL

ALL, acute lymphoblastic leukemia; AML, acute myeloid leukemia; and MDS, myelodysplastic syndrome.

## References

[1] Gratwohl A, Baldomero H, Aljurf M, Pasquini MC, Bouzas LF, Yoshimi A (2010). Hematopoietic stem cell transplantation: a global perspective. JAMA.

[2] Gratwohl A, Pasquini MC, Aljurf M, Atsuta Y, Baldomero H, Foeken L (2015). One million haemopoietic stem-cell transplants: a retrospective observational study. Lancet Haematol.

[3] Passweg JR, Baldomero H, Bregni M, Cesaro S, Dreger P, Duarte RF (2013). Hematopoietic SCT in Europe: data and trends in 2011. Bone Marrow Transplant.

[4] Rodriguez-Galindo C, Friedrich P, Alcasabas P, Antillon F, Banavali S, Castillo L (2015). Toward the Cure of All Children With Cancer Through Collaborative Efforts: Pediatric Oncology As a Global Challenge. J Clin Oncol.

[5] D'Souza A, Lee S, Zhu X, Pasquini M (2017). Current Use and Trends in Hematopoietic Cell Transplantation in the United States. Biol Blood Marrow Transplant.

[6] Sun CL, Francisco L, Kawashima T, Leisenring W, Robison LL, Baker KS (2010). Prevalence and predictors of chronic health conditions after hematopoietic cell transplantation: a report from the Bone Marrow Transplant Survivor Study. Blood.

[7] Skinner R, Wallace WH, Levitt GA, UK Children's Cancer Study Group Late Effects Group (2006). Long-term follow-up of people who have survived cancer during childhood. Lancet Oncol.

[8] Shenoy S, Angelucci E, Arnold SD, Baker KS, Bhatia M, Bresters D (2017). Current Results and Future Research Priorities in Late Effects after Hematopoietic Stem Cell Transplantation for Children with Sickle Cell Disease and Thalassemia: A Consensus Statement from the Second Pediatric Blood and Marrow Transplant Consortium International Conference on Late Effects after Pediatric Hematopoietic Stem Cell Transplantation. Biol Blood Marrow Transplant.

[9] Nakagawa R, Hosokawa-Tsuji A, Aoki Y, Takasawa K, Maru M, Nakajima K (2018). Total body irradiation for hematopoietic stem cell transplantation during early childhood is associated with the risk for diabetes mellitus. Endocrine.

[10] Frascino AV, Costa C, Salgado DMRA, Coracin FL, Fava M, Odone-Filho V (2019). Mandibular radiomorphometric assessment of bone mineral density in survivors of pediatric hematopoietic stem-cell transplantation. Clinics.

[11] Gurney JG, Kaste SC, Liu W, Srivastava DK, Chemaitilly W, Ness KK (2014). Bone mineral density among long-term survivors of childhood acute lymphoblastic leukemia: results from the St. Jude Lifetime Cohort Study. Pediatr Blood Cancer.

[12] Tanzi EM (2011). Health-related quality of life of hematopoietic stem cell transplant childhood survivors: state of the science. J Pediatr Oncol Nurs.

[13] Frascino AV, Fava M, Filho VO (2016). Short and long-term oral health-related quality of life perception in childhood onco-hematological cancer. Revista CPAQV.

[14] Frascino AV, Coracin FL, Santos PSS (2014). A systematic review of the long-term effects of dental development disturbances after hematopoietic stem-cell transplantation in pediatric patients. Clin Lab Res Den.

[15] Nishimura S, Inada H, Sawa Y, Ishikawa H (2013). Risk factors to cause tooth formation anomalies in chemotherapy of paediatric cancers. Eur J Cancer Care.

[16] Vesterbacka M, Ringden O, Remberger M, Huggare J, Dahllof G (2012). Disturbances in dental development and craniofacial growth in children treated with hematopoietic stem cell transplantation. Orthod Craniofac Res.

[17] Lemos AD, Katz CR, Heimer MV, Rosenblatt A (2014). Mandibular asymmetry: a proposal of radiographic analysis with public domain software. Dental Press J Orthod.

[18] Maber M, Liversidge HM, Hector MP (2006). Accuracy of age estimation of radiographic methods using developing teeth. Forensic Sci Int.

[19] Gawade PL, Hudson MM, Kaste SC, Neglia JP, Wasilewski-Masker K, Constine LS (2014). A systematic review of selected musculoskeletal late effects in survivors of childhood cancer. Curr Pediatr Rev.

[20] Al-Jewair TS, Preston CB, Flores-Mir C, Ziarnowski P (2018). Correlation between craniofacial growth and upper and lower body heights in subjects with Class I occlusion. Dental Press J Orthod.

[21] Sanders JE (2008). Growth and development after hematopoietic cell transplant in children. Bone Marrow Transplant.

[22] Huang YS, Hsu JF, Paiva T, Chin WC, Chen IC, Guilleminault C (2019). Sleep-disordered breathing, craniofacial development, and neurodevelopment in premature infants: a 2-year follow-up study. Sleep Med.

[23] Calvo-Henriquez C, Martins-Neves S, Faraldo-Garcia A, Ruano-Ravina A, Rocha S, Mayo-Yanez M (2019). Are pediatricians and otolaryngologists well prepared to identify early signs of vertical facial growth?. Int J Pediatr Otorhinolaryngol.

[24] Ruyssinck L, Toulouse K, Bordon Cueto de Braem V, Cauwels R, Dhooge C (2019). Impact of Hematopoietic Stem Cell Transplantation on Dental Development. Biol Blood Marrow Transplant.

